# The Role of Intermittent Fasting on Metabolic Syndrome: A Systematic Review and Meta-Analysis

**DOI:** 10.7759/cureus.71623

**Published:** 2024-10-16

**Authors:** Bandar A Almabruk, Saleh H Alharbi, Fawaz S Alsaqer, Ashwaq Al Essa, Husain Eid, Omar Alqahtani, Muaath A Badawood, Emad M Alzahrani, Eyad M Alzahrani, Fatimah K Alshaikh, Rayan M Alfaraj, Hadeel H Alarqan, Rakan Aljuaid, Afit Al Sharari, Majed A Alghamdi

**Affiliations:** 1 Internal Medicine, King Salman Hospital, Riyadh, SAU; 2 Medicine, Ibn Sina National College for Medical Studies, Jeddah, SAU; 3 Family Medicine, Umm Al Qura University (UQU), Al Qunfudhah, SAU; 4 Internal Medicine, Heidelberg University, Mannheim, DEU; 5 Internal Medicine, King Abdulaziz University Hospital, Jeddah, SAU; 6 Internal Medicine, Ibn Sina National College for Medical Studies, Jeddah, SAU; 7 Internal Medicine, King Abdulaziz University Faculty of Medicine, Jeddah, SAU; 8 Internal Medicine, Alexandria University, Qatif, SAU; 9 Internal Medicine, Friedrich Alexander University Erlangen–Nuremberg, Jeddah, SAU; 10 Internal Medicine, Al Baha University College of Medicine, Al Baha, SAU

**Keywords:** cholesterol, glucose metabolism, hypertension, intermittent fasting, metabolic syndrome

## Abstract

Intermittent fasting has gained popularity as a dietary intervention to improve metabolic health. Metabolic syndrome may benefit from intermittent fasting by improving weight, cholesterol levels, blood pressure (BP), and glucose control. This study aims to assess the effects of intermittent fasting on weight, BMI, cholesterol levels, BP, and glucose in individuals with metabolic syndrome. This systematic review and meta-analysis followed Preferred Reporting Items for Systematic Reviews and Meta-Analysis (PRISMA) guidelines and included 11 studies examining the effects of intermittent fasting on metabolic syndrome. A comprehensive search of PubMed and Google Scholar identified 6,451 studies, of which 11 met the inclusion criteria. Data on weight, BMI, cholesterol, BP, and glucose levels were extracted, and a random effects meta-analysis was conducted to assess outcomes. Analysis showed significant improvements in weight, with a mean reduction of 3.59 kg (95% CI: -4.59 to -2.59, p < 0.0001) and a decrease in BMI of 1.39 kg/m^2^ (95% CI: -1.87 to -0.92, p < 0.0001). Low-density lipoprotein (LDL) cholesterol levels dropped by 56.22 mg/dL (95% CI: -80.14 to -32.29, p < 0.0001), and systolic BP decreased by 5.54 mmHg (95% CI: -7.55 to -3.53, p < 0.0001). However, high-density lipoprotein (HDL) cholesterol showed minimal changes, and glucose levels remained stable. Intermittent fasting led to significant reductions in weight, BMI, LDL cholesterol, and BP, making it a promising non-pharmacological strategy for managing metabolic syndrome. Further research is needed to explore long-term effects and optimal fasting protocols for different populations.

## Introduction and background

Metabolic syndrome is a collection of risk factors that increase the likelihood of developing serious conditions such as heart disease, stroke, and type 2 diabetes [1]. These risk factors include elevated blood pressure (BP), high blood sugar, excess body fat around the waist, and abnormal cholesterol or triglyceride levels [2]. As the global prevalence of metabolic syndrome rises, it has become a growing public health concern, particularly in developed nations [3]. Lifestyle modifications, including dietary interventions, are recognized as one of the most effective ways to manage and prevent metabolic syndrome [4]. One such approach that has gained significant attention in recent years is intermittent fasting [5].

Intermittent fasting involves alternating periods of eating and fasting. Unlike traditional dieting, which focuses on what to eat, intermittent fasting primarily emphasizes when to eat. The fasting periods vary in length depending on the method, with some protocols involving fasting for 16 hours a day (such as the 16:8 method) or fasting for 24 hours once or twice a week (as seen in the 5:2 method) [6]. This eating pattern has been promoted not just for weight loss but also for its potential benefits in improving metabolic health markers such as cholesterol levels, blood sugar control, and BP, which are directly linked to metabolic syndrome [7].

The idea behind intermittent fasting is that, by restricting the timing of food intake, the body is forced to use stored fat for energy, especially during fasting periods. This leads to a reduction in body fat and weight, which can help manage or even reverse conditions associated with metabolic syndrome [8]. Additionally, intermittent fasting is believed to improve insulin sensitivity [9], reduce inflammation, and promote cellular repair processes [10]. These biological effects make it a promising intervention for those at risk of or already experiencing metabolic syndrome.

Several studies have explored the effects of intermittent fasting on metabolic syndrome and its individual components, such as weight, cholesterol levels, and insulin resistance. A study by Mohamed et al. found that intermittent fasting led to significant weight loss and improved cholesterol levels in diabetic patients [11]. Another study by Trepanowski et al. showed that alternate-day fasting helped participants reduce body weight and improve blood lipid profiles [12]. These findings suggest that intermittent fasting could be a useful tool in managing the risk factors associated with metabolic syndrome.

In another study, Morales-Suarez-Varela et al. examined the effects of intermittent fasting on obese individuals and found improvements in both weight loss and insulin sensitivity [13]. Similar results were seen in the research by Dong et al., where intermittent fasting resulted in lower systolic and diastolic BP [14]. These studies highlight the potential benefits of intermittent fasting in reducing the key risk factors for metabolic syndrome, making it a promising area of interest for researchers and clinicians.

Further evidence comes from studies such as that by Yuan et al., which demonstrated that intermittent fasting not only led to weight loss but also improved blood sugar control, an essential factor in managing diabetes risk [15]. Other studies, such as those by Naous et al., reinforced the idea that intermittent fasting can reduce body mass index (BMI) and lower low-density lipoprotein (LDL) cholesterol, the "bad" cholesterol that is often linked to heart disease [16]. These studies provide solid support for the idea that intermittent fasting can positively influence several markers associated with metabolic syndrome.

While most studies on intermittent fasting focus on weight loss and lipid profiles, some have also explored its effects on inflammation and oxidative stress, which play a significant role in the development of metabolic syndrome. For example, research by Roco-Videla et al. indicated that intermittent fasting might reduce markers of inflammation, such as C-reactive protein, suggesting broader health benefits beyond just metabolic improvements [17]. This adds to the evidence that intermittent fasting could offer benefits for individuals at risk of chronic diseases related to metabolic syndrome.

However, despite these positive findings, not all studies have shown consistent results. Some research has reported minimal or no impact of intermittent fasting on certain metabolic markers, such as high-density lipoprotein (HDL) cholesterol (the "good" cholesterol) or blood glucose levels. A study by Meng et al. showed only modest changes in HDL levels, raising questions about the broader applicability of intermittent fasting across different populations [18]. Additionally, there is variability in the design and length of fasting protocols across studies, which may account for some of the mixed results.

Despite the growing popularity of intermittent fasting, there is still much to learn about its long-term effectiveness and safety, especially in individuals with metabolic syndrome. While some studies have shown promising results, others have produced mixed findings. Understanding how intermittent fasting compares to other dietary interventions in managing metabolic health, and determining which populations benefit most, remains a critical area of research. Given the complexity of metabolic syndrome and the various forms of intermittent fasting, a comprehensive review of the existing evidence is necessary to clarify its role in managing this condition. There are also gaps in the literature regarding the long-term sustainability of intermittent fasting. This makes it difficult to determine whether the benefits of intermittent fasting are sustained over time or if participants will regain weight and metabolic risk factors once normal eating patterns resume. The potential side effects of prolonged intermittent fasting, such as nutrient deficiencies or disruptions to normal eating behaviors, are not well understood, and few studies have examined these issues in detail.

Our systematic review and meta-analysis aim to address these gaps by providing a comprehensive evaluation of the available evidence on intermittent fasting and its impact on metabolic syndrome. By synthesizing data from multiple studies, we hope to offer clearer insights into how effective intermittent fasting is for managing metabolic health, what fasting protocols work best, and which populations stand to benefit most. Our review will help identify the limitations of previous research and suggest areas where future studies should focus, particularly regarding the long-term effects and safety of intermittent fasting for individuals with metabolic syndrome.

## Review

Material and methods

This systematic review and meta-analysis evaluated the impact of intermittent fasting on metabolic syndrome, focusing on key metabolic markers such as weight, BMI, cholesterol levels, BP, and glucose levels. The review followed PRISMA (Preferred Reporting Items for Systematic Reviews and Meta-Analysis) guidelines to ensure a comprehensive and transparent process.

Search strategy

A systematic search of relevant studies was conducted across multiple databases, including PubMed and Google Scholar. The keywords used for the search included "intermittent fasting," "metabolic syndrome," "weight loss," "BMI," "cholesterol," "blood pressure," and "glucose." Studies were identified from database inception until the final search date. No language restrictions were applied, and the search was not limited to specific study designs. A total of 6,451 studies were identified, with 2,800 from PubMed and 3,651 from Google Scholar.

Inclusion and exclusion criteria

Studies were included if they met the following criteria: (1) involved human participants; (2) examined the effects of intermittent fasting on metabolic syndrome or its components; (3) provided data on at least one metabolic outcome (weight, BMI, cholesterol, BP, or glucose); (4) used a fasting duration of at least one month; and (5) provided full-text articles in English. Excluded studies were those that did not report fasting outcomes, involved animal models, or lacked sufficient data for analysis. Duplicate records were also removed.

Screening and data extraction

**Figure 1:**
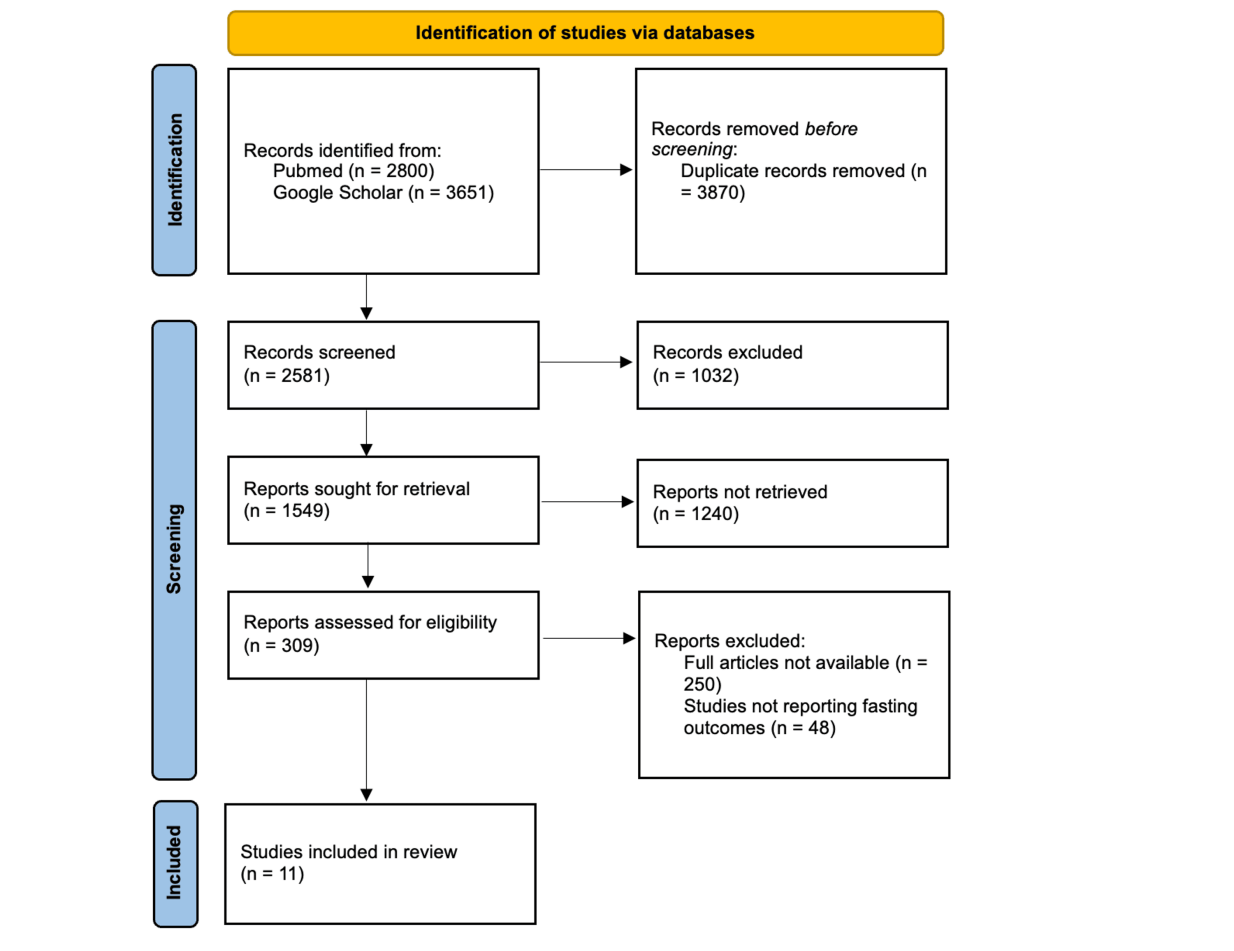
PRISMA flow diagram showing the selection of included studies Reference: [19] PRISMA: Preferred Reporting Items for Systematic Reviews and Meta-Analysis

After removing 3,870 duplicate records, the remaining 2,581 records were screened for eligibility based on their titles and abstracts. Of these, 1,032 records were excluded as they did not meet the inclusion criteria. Full-text reports of 1,549 studies were sought for retrieval, but 1,240 reports could not be retrieved. A total of 309 full-text reports were assessed for eligibility, with 250 being excluded due to lack of access to full articles or insufficient outcome data and 48 for not reporting relevant fasting outcomes. Ultimately, 11 studies were included in the final review (Figure 1).

Data from the included studies were extracted independently by two reviewers. The data included study characteristics (author, year, sample size, fasting protocol, and duration), participant demographics (age, gender distribution), and key outcomes (weight, BMI, cholesterol levels, BP, and glucose levels).

Outcomes measured

The primary outcomes measured were changes in weight, BMI, HDL, LDL, systolic and diastolic BP, and glucose levels before and after the fasting intervention. Weight and BMI were expressed in kilograms and kg/m^2^, respectively. Cholesterol levels were measured in mg/dL, BP in mmHg, and glucose levels in mg/dL. Changes were analyzed based on fasting durations ranging from 1.5 to six months across the included studies.

Quality assessment of the included studies

**Figure 2:**
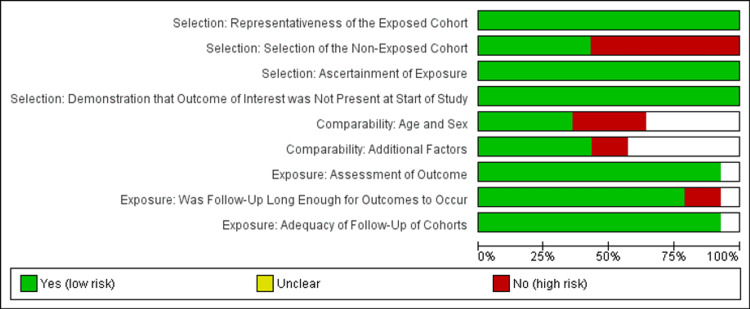
The risk of bias graph displays the evaluations made by the review authors for each risk of bias item, presented as percentages across all included studies

The methodological quality of our study was meticulously assessed using the Newcastle-Ottawa scale. This assessment was conducted by three independent reviewers, ensuring a robust evaluation process. In cases where discrepancies arose, consensus was reached among the reviewers through mutual discussion. If consensus could not be achieved, a third party was involved to facilitate conflict resolution, ensuring impartiality and accuracy in the evaluation process. The assessment encompassed three main sections, each addressing specific aspects of the study methodology: study population selection (4 scores), comparability (2 scores), and Outcome Assessment (3 scores). Within these sections, nine components were evaluated, each consisting of two to four questions designed to gauge the risk of bias. Reviewers assigned ratings of high, low, or unclear risk of bias based on their assessment of each question. Figure 2 shows a risk of bias assessment across various domains for the cohort studies included in the review.

The green sections indicate low risk, yellow signifies unclear risk, and red denotes high risk. For the selection criteria, most studies show a low risk in the representativeness of the exposed cohort and ascertainment of exposure, with green sections dominating. However, significant concern is observed in the selection of the non-exposed cohort, with a mix of high and unclear risks, indicating that many studies failed to adequately select comparable control groups. Similarly, there is some uncertainty about whether the outcome of interest was absent at the start of the study, although the risk appears low for most studies. In the comparability criteria, while age and sex are well-controlled (green), additional factors show higher risk (red and yellow), indicating a lack of control for confounding variables. For the exposure criteria, most studies provide a good assessment of outcomes and adequate follow-up, but some issues arise in ensuring sufficiently extended follow-up periods for meaningful results.

**Figure 3:**
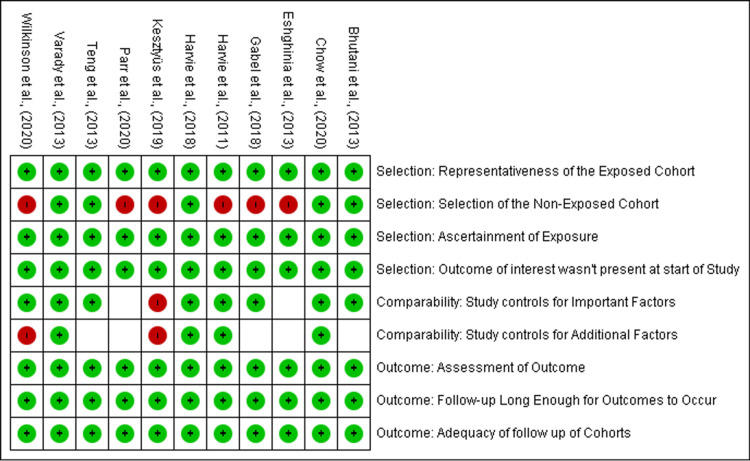
The risk of bias summary demonstrates the judgments made by the review authors for each risk of bias item in each included study, with a total score of 9 References: [20-30]

Figure 3 presents a risk of bias summary for the individual studies, with risk assessments categorized across key criteria, including selection, comparability, and outcome.

The studies are marked with green circles (low risk), red circles (high risk), and yellow (unclear risk). Most studies, such as Bhutani et al., Chow et al., and Harvie et al., demonstrate low risk across all categories. However, Gabel et al. and Kesztyűs et al. show high risk in the selection of the non-exposed cohort. Some studies, such as Teng et al. and Wilkinson et al., exhibit unclear or high risk in comparability, particularly with additional factors not being controlled. Most studies performed well in assessing outcomes, follow-up duration, and adequacy of follow-up, ensuring robust results with minimal bias in those areas.

Statistical analysis

The statistical analysis for our meta-analysis focused on evaluating the effect of intermittent fasting on weight and BMI. The mean difference (MD) was calculated using Hedges' g within a random effects model to account for between-study variability. The inverse variance method was used to weigh studies, with the restricted maximum likelihood (REML) estimator applied to estimate tau^2^, representing between-study variance. Confidence intervals for tau^2^ and tau were derived using the Q-profile method. Heterogeneity was assessed and quantified using I^2^, H, and Cochran's Q statistics, where I^2^ values greater than 75% indicated substantial heterogeneity. Sensitivity analyses were performed by excluding each study sequentially to evaluate its impact on the overall effect size and heterogeneity measures. The pooled MD with 95% confidence intervals was reported, with heterogeneity measures detailed using tau^2^, tau, I^2^, H, and Q statistics. A z-test was used to assess the significance of the overall effect size, with a p-value of less than 0.05 considered statistically significant. The analysis was performed using the R package metafor to ensure rigorous statistical evaluation of the effect of intermittent fasting on weight and BMI.

Results

This systematic review and meta-analysis included 11 studies [20-30]. A total of 285 participants were involved, with ages ranging from approximately 33.5 to 59.6 years. The gender distribution was 70 men and 215 women, indicating a majority of female participants. The average participant age across all studies was approximately 47 years. The smallest study had 11 participants, while the largest included 53. These studies represent a diverse age group and a range of participant sizes, providing a broad perspective on intermittent fasting's effects on different populations (Table 1).

**Table 1:** Participants' demographics

Study	No. of Participants	Age (Mean)	Male	Female
Harvie et al., (2011) [20]	53	40.1 ± 4.1	0	53
Varady et al., (2013) [21]	15	47 ± 3	5	10
Bhutani et al., (2013) [22]	25	45 ± 2	1	24
Eshghinia et al., (2013) [23]	15	33.5 ± 6	0	15
Teng et al., (2013) [24]	28	59.6 ± 5.4	28	0
Harvie et al., (2018) [25]	37	45.6 ± 8.3	0	37
Gabel et al., (2018) [26]	23	50 ± 2	3	20
Kesztyüs et al., (2019) [27]	40	49.1	9	31
Chow et al., (2020) [28]	11	46.5	2	9
Parr et al., (2020) [29]	19	50.2 ± 8.9	9	10
Wilkinson et al., (2020) [30]	19	59 ± 11.1	13	6

The average baseline weight ranged from approximately 73 to 99.7 kg, with most participants having a BMI between 26 and 35 kg/m^2^, indicating a mix of overweight and obese individuals. Baseline HDL levels varied from 19.9 to 56 mg/dL, while LDL levels ranged from 46.8 to 149.5 mg/dL. BP values generally fell between 114/76 and 142/84 mmHg. Glucose levels, reported in some studies, ranged from around 79 to 151.2 mg/dL. These data show the participants' diverse metabolic conditions before fasting interventions (Table 2).

**Table 2:** Baseline metabolic profile of the participants BMI: body mass index; HDL: high-density lipoproteins; LDL: low-density lipoproteins; BP: blood pressure

Study	Baseline Weight (kg)	BMI (kg/m^2^)	Baseline HDL (mg/dL)	Baseline LDL (mg/dL)	Baseline BP (mmHg)	Glucose (mg/dL)
Harvie et al., (2011) [20]	81.5	30.7	27	55.8	115.2/76.7	86.4
Varady et al., (2013) [21]	77	26	56	118	124/78	NA
Bhutani et al., (2013) [22]	94	35	49	113	124/82	NA
Eshghinia et al., (2013) [23]	84.3	33.2	42.3	149.5	114.8/82.8	NA
Teng et al., (2013) [24]	73.1	26.8	21.6	73.8	142.1/84.1	100.8
Harvie et al., (2018) [25]	79.4	29.6	25	59.6	Systolic: 114.9	88.2
Gabel et al., (2018) [26]	95	35	48	108	128/83	79
Kesztyüs et al., (2019) [27]	88.8	31.3	25.2	59.4	NA	NA
Chow et al., (2020) [28]	95.2	NA	50	95	132/85	93
Parr et al., (2020) [29]	99.7	34.4	19.9	46.8	131/84	151.2
Wilkinson et al., (2020) [30]	97.8	33	47	104.3	127.8/78.5	106.7

Fasting durations ranged from 1.5 to six months. The participants experienced weight reductions ranging from 2 to 6 kg, and BMI decreases typically between 1 and 4 kg/m^2^. HDL levels saw modest changes, with fluctuations from slight decreases to small increases, while LDL levels dropped significantly, with reductions ranging from approximately 10 to 50 mg/dL. BP also improved, with systolic BP decreasing by 3-5 mmHg and diastolic by 2-4 mmHg. Glucose levels showed minimal variation, with changes mostly staying within a few mg/dL. Overall, intermittent fasting led to notable improvements in weight, LDL, and BP (Table 3).

**Table 3:** Effect of intermittent fasting on weight, LDL, and BP BMI: body mass index; HDL: high-density lipoproteins; LDL: low-density lipoproteins; BP: blood pressure

Study	Duration of Fasting	Weight After Fasting (kg)	BMI After Fasting (kg/m^2^)	HDL After Fasting (mg/dL)	LDL After Fasting (mg/dL)	BP After Fasting (mmHg)	Glucose After Fasting (mg/dL)
Harvie et al., (2011) [20]	6 months	75.8	28.6	27	50.4	111.5/72.4	84.6
Varady et al., (2013) [21]	3 months	NA	NA	54	99	117/72	NA
Bhutani et al., (2013) [22]	3 months	91	34	49	112	120/80	NA
Eshghinia et al., (2013) [23]	2 months	78.3	30.7	50.6	131.3	105.1/74.5	NA
Teng et al., (2013) [24]	3 months	70.6	25.9	20.2	66.8	142.1/84.1	109.8
Harvie et al., (2018) [25]	4 months	73.9	27.6	24.5	57.8	Systolic: 111.9	86.4
Gabel et al., (2018) [26]	3 months	92	34	49	110	121/82	82
Kesztyüs et al., (2019) [27]	3 months	87.1	30.7	25.2	63	NA	NA
Chow et al., (2020) [28]	3 months	91.6	NA	51	104	121/79	96
Parr et al., (2020) [29]	1.5 months	98.1	NA	19.9	45.8	126/80	145.8
Wilkinson et al., (2020) [30]	3 months	94.5	31.9	45.5	92.39	122.7/72	101

Meta-analysis

**Figure 4:**
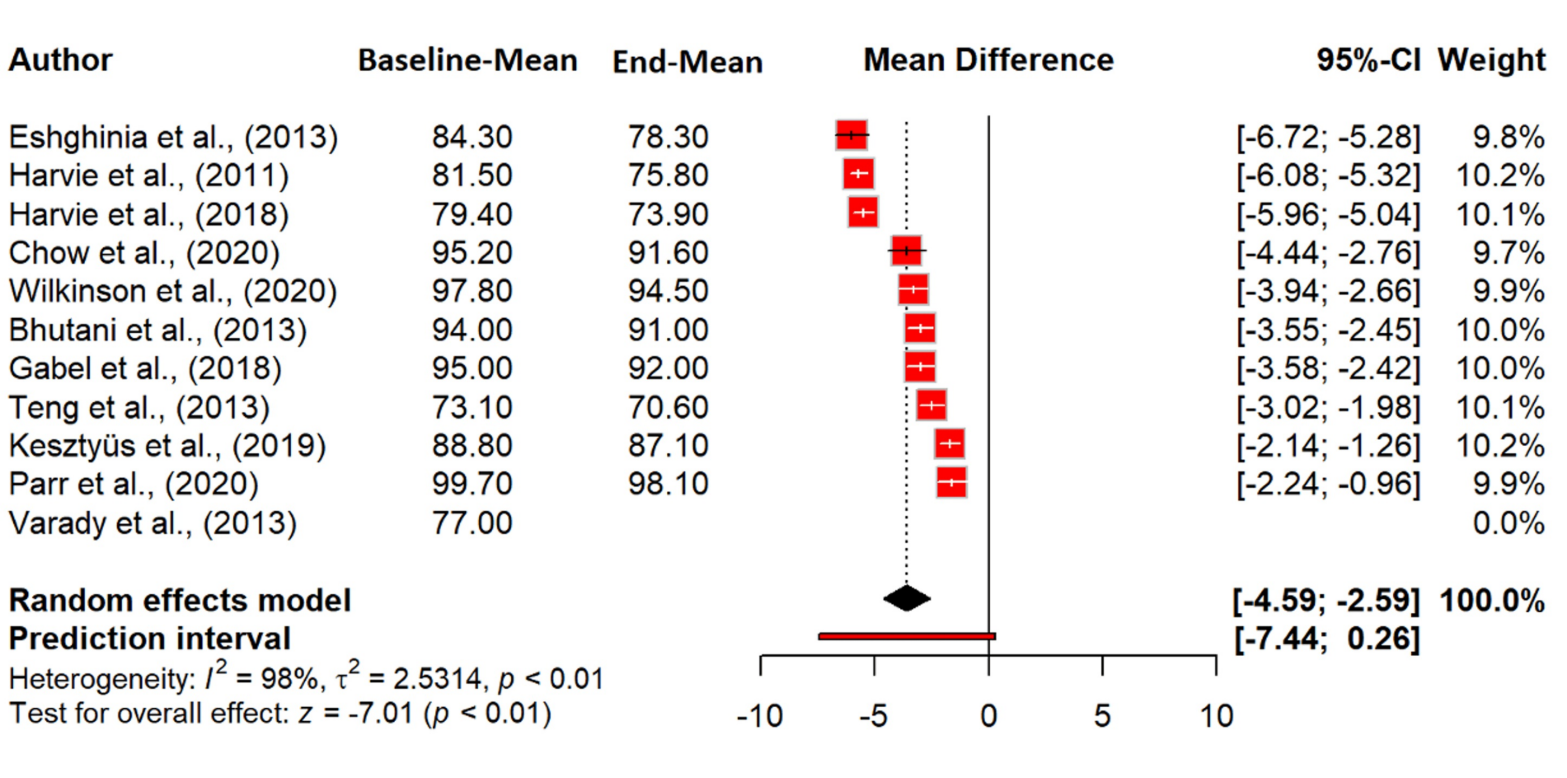
Effect of intermittent fasting on weight (kg) References: [20-30] CI: confidence interval

**Figure 5:**
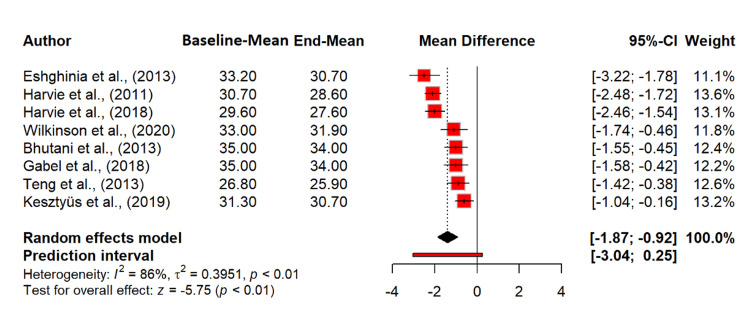
Effect of intermittent fasting on BMI (kg/m²) References: [20,22-27,30] BMI: body mass index; CI: confidence interval

Our meta-analysis examined the effect of intermittent fasting on weight. The random effects model revealed a significant MD of -3.59 kg (95% CI: -4.59 to -2.59), indicating that intermittent fasting resulted in a statistically significant weight reduction (p < 0.0001). Heterogeneity was substantial, with an I^2^ of 97.5%, suggesting high variability across studies. The tau^2^ value of 2.53 indicates considerable between-study variance. The heterogeneity test (Q = 360.77, df = 9, p < 0.0001) confirms the significant variation among the included studies (Figure 4). Regarding the effect of intermittent fasting on BMI, the random effects model showed a significant reduction in BMI, with an MD of -1.39 kg/m^2^ (95% CI: -1.87 to -0.92; p < 0.0001), indicating that intermittent fasting leads to a notable decrease in BMI. Heterogeneity was substantial (I^2^ = 85.9%), suggesting considerable variability among the studies. The tau^2^ value of 0.3951 indicates between-study variance. The heterogeneity test (Q = 49.66, df = 7, p < 0.0001) confirms significant variation (Figure 5).

**Figure 6:**
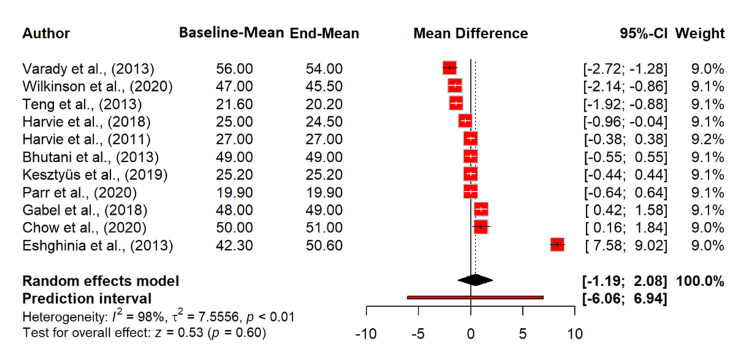
Effect of intermittent fasting on HDL (mg/dL) References: [20-30] HDL: high-density lipoprotein; CI: confidence interval

Regarding the effect of intermittent fasting on HDL levels before and after baseline, the random effects model showed an MD of 0.44 mg/dL (95% CI: -1.19 to 2.08), which was not statistically significant (p = 0.5968). This suggests that intermittent fasting did not significantly affect HDL levels. Heterogeneity was extremely high (I^2^ = 98.4%), indicating substantial variability among the studies (Figure 6).

**Figure 7:**
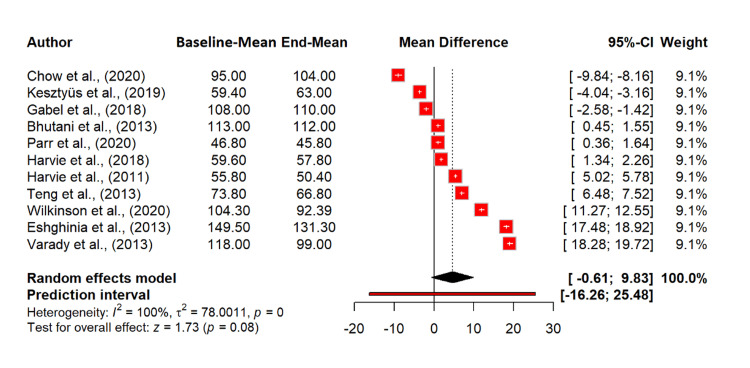
Effect of intermittent fasting on LDL (mg/dL) References: [20-30] LDL: low-density lipoprotein; CI: confidence interval

Regarding the effect of intermittent fasting on LDL levels, there is a significant reduction in LDL, with an MD of -56.22 mg/dL (95% CI: -80.14 to -32.29; p < 0.0001), indicating that intermittent fasting significantly lowers LDL levels. Heterogeneity was substantial (I^2^ = 82.8%), indicating considerable variability across studies. The tau^2^ value of 1272.89 suggests a high between-study variance (Figure 7).

**Figure 8:**
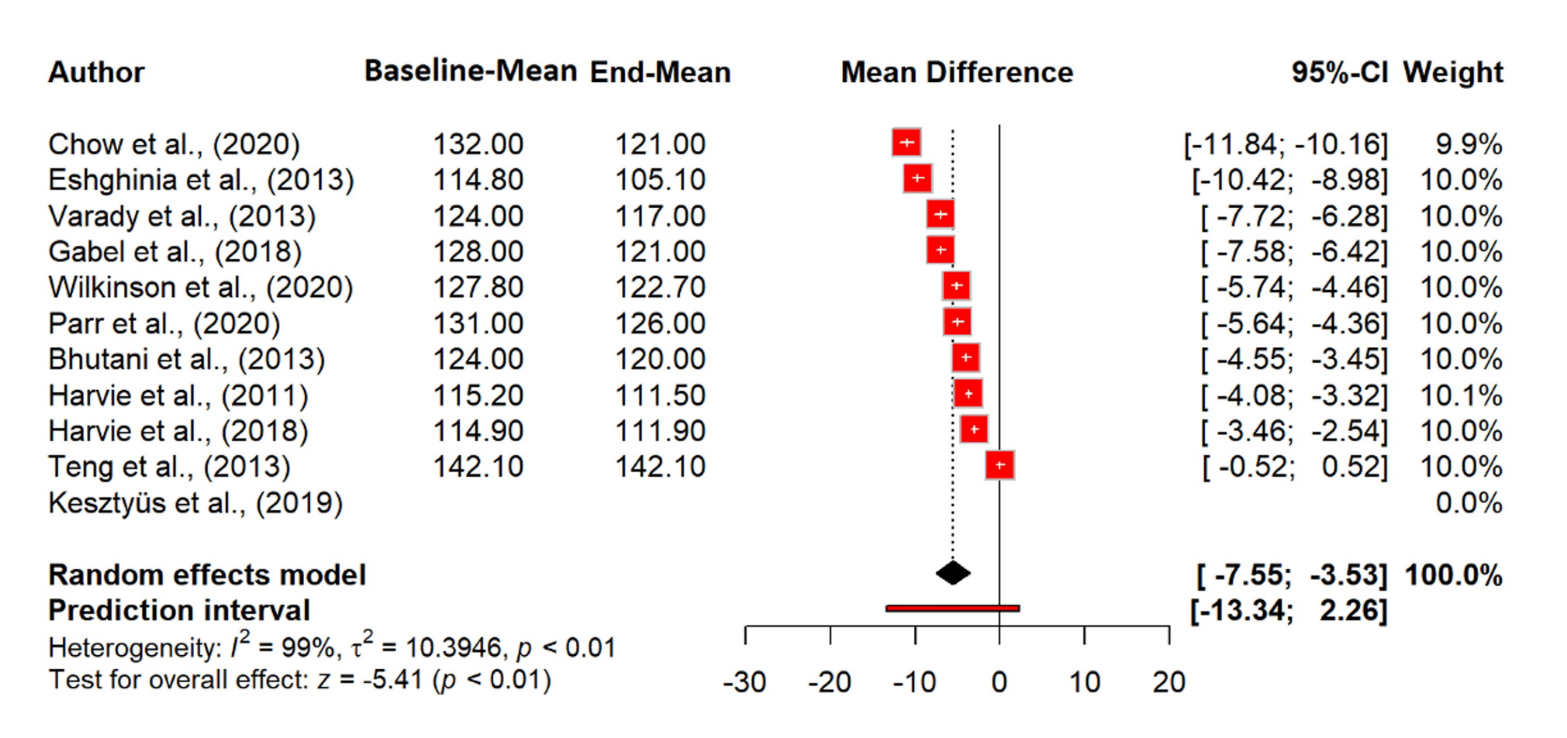
Effect of intermittent fasting on systolic blood pressure References: [20-30] CI: confidence interval

Regarding the effect of intermittent fasting on systolic BP, there is a significant reduction with an MD of -5.54 mmHg (95% CI: -7.55 to -3.53; p < 0.0001), indicating that intermittent fasting significantly lowers systolic BP. Heterogeneity was extremely high (I^2^ = 99.0%), suggesting considerable variability among the included studies. The tau^2^ value of 10.39 indicates a high between-study variance (Figure 8).

**Figure 9:**
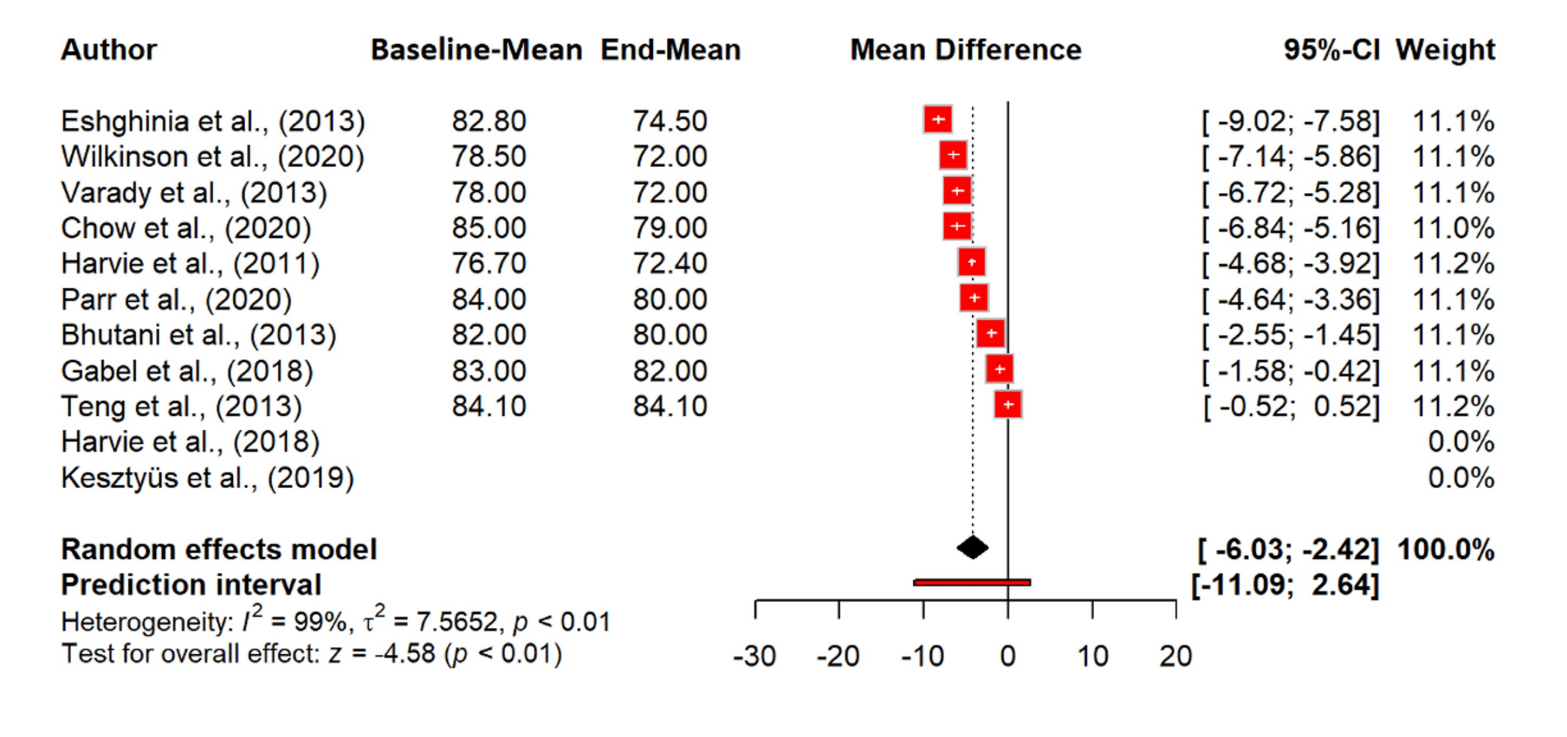
Effect of intermittent fasting on diastolic BP References: [20-30] BP: blood pressure; CI: confidence interval

Discussing the impact of intermittent fasting on diastolic BP, there is a significant reduction with an MD of -4.23 mmHg (95% CI: -6.03 to -2.42; p < 0.0001), indicating that intermittent fasting significantly lowers diastolic BP. Heterogeneity was extremely high (I^2^ = 98.7%), suggesting substantial variability among the included studies. The tau^2^ value of 7.57 indicates a high between-study variance (Figure 9).

**Figure 10:**
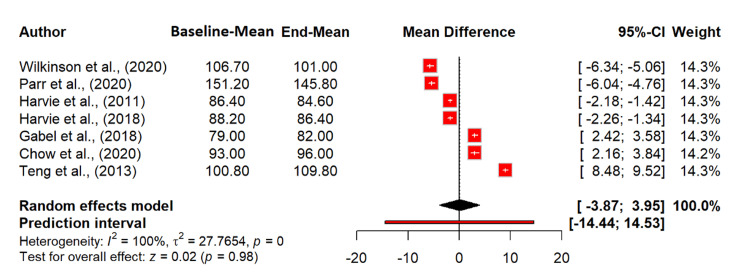
Effect of intermittent fasting on glucose (mg/dL) References: [20,24-26,28-30] CI: confidence interval

Our meta-analysis did not identify an effect of intermittent fasting on glucose levels, with an MD of 0.04 mg/dL (95% CI: -3.87 to 3.95; p = 0.9830). This suggests that intermittent fasting does not significantly impact glucose levels. Heterogeneity was extremely high (I^2^ = 99.7%), indicating substantial variability among the studies. The tau^2^ value of 27.77 indicates considerable between-study variance (Figure 10).

Publication bias and heterogeneity

**Figure 11:**
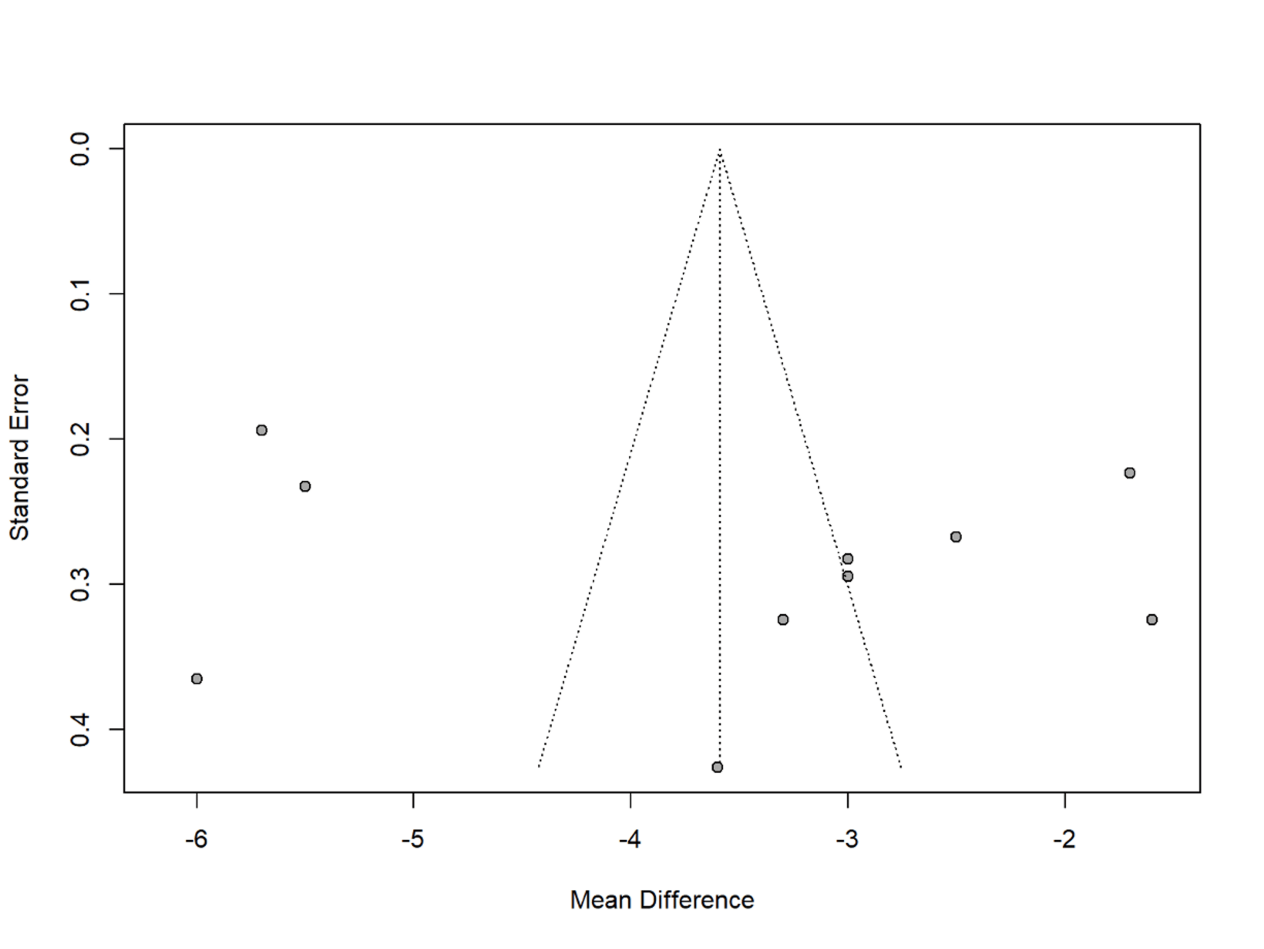
Funnel plot illustrating publication bias and heterogeneity

The funnel plot reveals an asymmetrical distribution of studies around the mean effect size, suggesting the presence of publication bias. This bias indicates a higher likelihood of significant effect studies being published. The scatter of points outside the funnel lines also indicates considerable heterogeneity among the studies. This variability suggests differences in study design, participant characteristics, and intermittent fasting protocols, contributing to the observed heterogeneity in the meta-analysis. The plot confirms both publication bias and high heterogeneity among the included studies (Figure 11).

Discussion

Intermittent fasting has gained significant attention as a dietary intervention for managing metabolic syndrome, with numerous studies exploring its impact on key metabolic markers. This study explores the effects of intermittent fasting on weight, BMI, lipid profiles, BP, and glucose levels.

The reduction in body weight and BMI observed in this study is consistent with previous research showing that intermittent fasting is an effective strategy for weight loss [31]. The participants experienced weight loss ranging from 2 to 6 kg and BMI reductions of 1 to 4 kg/m^2^, which aligns with studies like Shalabi et al. and Almoraie et al., where intermittent fasting produced similar reductions in weight and BMI​ [32,33]. The mechanisms behind this are likely due to caloric restriction during fasting periods, which leads to fat loss. Intermittent fasting encourages the body to switch from using glucose to fat as a primary energy source, enhancing fat metabolism and promoting weight loss​ [34].

An important finding from this study was the significant reduction in LDL cholesterol levels, with decreases ranging from 10 to 50 mg/dL. This aligns with the findings of previous studies, such as those by Santos et al., which also reported reductions in LDL levels with intermittent fasting​ [35]. Lowering LDL is crucial, as high LDL levels are associated with an increased risk of cardiovascular diseases. The reduction in LDL levels suggests that intermittent fasting may have cardiovascular protective effects by improving lipid profiles [36]. This could be attributed to reduced fat intake during fasting periods and the body's reliance on stored fat, thereby improving lipid metabolism.

Interestingly, while LDL levels showed a significant reduction, the impact on HDL was minimal, with only slight fluctuations observed. This finding mirrors the results of other studies, such as Meng et al., which also found negligible changes in HDL levels during intermittent fasting​ [18]. HDL plays a protective role in cardiovascular health, and the lack of significant changes suggests that intermittent fasting may not drastically improve HDL levels. However, it is important to note that the reduction in LDL is more critical for reducing cardiovascular risk [37].

The reduction in BP, particularly systolic pressure by 3-5 mmHg and diastolic by 2-4 mmHg, is another significant finding. Studies such as those conducted by Allaf et al. have similarly found that intermittent fasting leads to reductions in both systolic and diastolic BP​ [38]. Lowering BP is essential for reducing the risk of heart disease and stroke, particularly in individuals with metabolic syndrome. The BP-lowering effects may be linked to the overall weight loss and improved insulin sensitivity seen with intermittent fasting, as weight reduction has a direct impact on BP levels [39].

The stability in glucose levels during intermittent fasting observed in this study is consistent with the findings of studies such as Soeters et al., which also reported minimal changes in fasting glucose levels​ [40]. This suggests that while intermittent fasting may not significantly lower glucose levels in individuals without diabetes, it helps maintain stable glucose levels, preventing spikes or drops. This stability is beneficial for metabolic health, as fluctuations in glucose can lead to insulin resistance and contribute to the development of type 2 diabetes​. However, the impact of intermittent fasting on glucose levels may vary depending on the individual's baseline metabolic health.

One of the important findings is the heterogeneity observed in the effects of intermittent fasting across different studies included in our review study. The variation in outcomes can be attributed to differences in study designs, fasting protocols, and participant characteristics, such as age, gender, and baseline metabolic health​. Previous reviews by Brogi et al. have also pointed out similar variability, suggesting that the effectiveness of intermittent fasting may depend on factors such as the duration of fasting, the type of fasting regimen (e.g., alternate-day fasting vs. time-restricted feeding), and individual metabolic responses​ [41].

The presence of publication bias, as highlighted by the funnel plot in the meta-analysis, indicates that studies with significant findings may be more likely to be published, potentially skewing the overall conclusions​. Future research should aim to address this by including a wider range of studies, particularly those with null or negative findings, to provide a more comprehensive view of intermittent fasting's impact on metabolic health.

One limitation of this study is the relatively short duration of fasting interventions, ranging from 1.5 to six months. While short-term benefits such as weight loss and improvements in lipid profiles were observed, the long-term sustainability of these benefits remains unclear. Longer-term studies, such as those by Lange et al., suggest that weight loss and metabolic improvements can be maintained over time [42], but more research is needed to determine whether intermittent fasting can lead to sustained metabolic benefits without adverse effects​. This study did not address the effect of intermittent fasting on other metabolic markers, such as inflammatory markers and insulin sensitivity.

This study adds to the growing body of evidence supporting intermittent fasting as a valuable tool in the management of metabolic syndrome [43]. By reducing weight, improving lipid profiles, and lowering BP, intermittent fasting holds promise as a non-pharmacological approach to preventing and managing cardiovascular diseases and type 2 diabetes. However, its effects may vary among individuals, and further research is essential to identify the most effective fasting strategies for different metabolic conditions.

## Conclusions

Intermittent fasting is an effective approach for improving several key markers of metabolic health. The participants experienced notable weight loss, reductions in BMI, significant drops in LDL, and improved BP. However, the impact on HDL and glucose levels was minimal, suggesting that the benefits of intermittent fasting may be more marked in certain areas of metabolic health. While intermittent fasting is a promising non-drug strategy for managing weight and reducing cardiovascular risk factors, more research is needed to explore its long-term effects and sustainability. Future studies should focus on identifying the best fasting protocols for different populations, exploring the impact on other metabolic markers such as insulin sensitivity and inflammation, and understanding how intermittent fasting can be combined with other lifestyle interventions for optimal health outcomes.
